# Updating the genomic and clinicopathologic features of thoracic SMARCA4-deficient undifferentiated tumor: a mini-series including a long-term survivor

**DOI:** 10.3389/fonc.2025.1601443

**Published:** 2025-08-20

**Authors:** Kenneth Ofori, Carlos Pagan, Marie C. Smithgall, Asma Salah Jadalla, Swikrity Upadhyay Baskota, John P. Crapanzano, Susan Hsiao, Mahesh M. Mansukhani

**Affiliations:** ^1^ Department of Pathology and Laboratory Medicine, Indiana University School of Medicine, Indianapolis, IN, United States; ^2^ Department of Pathology and Cell Biology, Columbia University Irving Medical Center, New York City, NY, United States; ^3^ Department of Pathology and Laboratory Medicine, University of California Davis Health System, Sacramento, CA, United States

**Keywords:** SMARCA4 deficiency, lung cancer, FL1, WT1, INSM1

## Abstract

**Introduction:**

Thoracic SMARCA4-deficient undifferentiated tumor (SMARCA4-dUT) is a recently described type of lung cancer, presenting as a bulky mass variably involving the mediastinum and the lung in patients with smoking history, and exhibits adverse prognosis. The essential diagnostic immunomorphologic features and typical genomic findings have been described. However, there is a continuing need to catalogue the spectrum of genomic changes underlying the disease, the heterogeneity of antigen expression in order to avoid diagnostic pitfalls, and any variability in patient outcomes. We sought to update the literature on the clinicopathologic and genomic characteristics of thoracic SMARCA4- dUT.

**Methods:**

We searched for cases diagnosed in our institution, reviewed clinical data, performed comprehensive genomic analysis, and evaluated immunomorphologic features.

**Results:**

Four cases (three males and one female) were identified at a median age of 61.5 years (range, 49–72 years), all with smoking history. The series included a patient with limited disease treated with surgery and adjuvant chemotherapy, who remained disease-free over a year after diagnosis, underscoring the importance of lung cancer screening among smokers and the possibility of a subgroup of thoracic SMARCA4-dUT with less aggressive disease. In addition to the known immunophenotypic features of the disease, we identified the expression of FLI (in three out of three cases) and WT-1 (in one of three cases), which are endothelial and mesothelial markers, and are findings to be cognizant of to avoid misdiagnosis as angiosarcoma or mesothelioma, respectively. While the neuroendocrine markers synaptophysin and CD56 were variably expressed in some cases, the expression of INSM1 was absent in all cases. Genomic analysis demonstrated tobacco-related features, including a high median tumor mutation burden and *TP53* variants. In this limited series, mutational signature analysis revealed evidence of SBS87 as the predominant single-base substitution COSMIC signature.

**Conclusion:**

Our work expands the possible diagnostic antigen expression of thoracic SMARCA4-dUT, contributes to the emerging reports on patients with variant disease presentation, and highlights the need for large-scale genomic studies to determine additional mechanisms of the initiation of carcinogenesis.

## Introduction

Thoracic SMARCA4-deficient undifferentiated tumor (SMARCA4-dUT) is a recently defined high-grade malignancy involving the thorax (1–3). The disease shares overlapping features with the relatively more common SMARCA4-deficient non-small cell lung carcinoma (SMARCA4-dNSCC) (3, 4). In addition to SMARCA4-dNSCC, the clinicoradiologic, morphologic, and/or immunophenotypic features of thoracic SMARCA4-dUT are similar to those of other malignancies including neuroendocrine carcinomas, mesotheliomas, and some sarcomas (3–5). While the defining morphologic and immunophenotypic features of thoracic SMARCA4-dUT have been established, it is imperative to delineate its full spectrum of immunohistochemical marker expression to ensure accurate differentiation from related entities.

Thoracic SMARCA4-dUT is rapidly progressive, usually presenting at an advanced stage, and is thought to uniformly show poor prognosis, with a median survival of 4–7 months (2, 6–9). Reports of cases presenting with limited disease and some durable response to therapy are, however, emerging (10–15). Comprehensive genomic characterization enables the identification of gene variants and genomic signatures with possible insights into the initiators of carcinogenesis and variability in the clinical course. A few studies have comprehensively evaluated the genomic features of thoracic SMARCA4-dUT, but the genomic landscape is still being defined (4, 7).

In this study, we seek to update the literature on the spectrum of clinical, immunophenotypic, and genomic findings in thoracic SMARCA4-dUT.

## Methods

### Clinical and pathological data

This is a retrospective single-institution study. The database of the Pathology Department of the Columbia University Irving Medical Center (CUIMC) was searched for cases of thoracic SMARCA4-dUT diagnosed from January 2021 to January 2023. Cases of SMARCA4-dNSCC were excluded. Available data regarding the clinical presentation, radiologic features, treatment modalities, and outcomes were retrieved from the electronic medical records. The study was conducted according to the Helsinki Declaration.

### Immunohistochemistry

An extensive panel of immunohistochemical staining was performed based on the availability of tissue for testing. The cases were assessed for the expression of SMARCA4 (BRG1), SMARCA2 (BRM), SMARCB1 (INI), pan-cytokeratin, claudin-4, CAM5.2, TTF-1, p40, p63, CK5, CK7, WT-1, calretinin, CK20, chromogranin, synaptophysin, CD56, INSM1, RB, NUT1, SOX2, OCT4, CD34, SALL4, FLI1, and CD10 together with those of other antibodies in an extensive panel to determine the lineage of neoplastic cells. **Supplementary Table S1** details the pertinent antibody clones and dilutions used.

### Next-generation sequencing and data analysis

A custom comprehensive panel detecting single nucleotide variants (SNVs), small insertions and deletions (indels), copy number variants (CNVs), and genomic rearrangements in 586 cancer-related genes, tumor mutation burden (TMB), and microsatellite instability was performed as previously described (16). Briefly, after microdissection to enrich for lesional cells, the genomic DNA obtained from the tumor was fragmented and amplified. Amplified libraries underwent positive selection using DNA probes targeting regions of interest. The selected library was further amplified, normalized, and loaded onto an Illumina platform (NextSeq2000, San Diego, CA, USA) for paired-end sequencing. In addition, following first- and second-strand cDNA synthesis, the cDNA was also used as a template for the same library preparation, enrichment, and sequencing processes as genomic DNA. Following sequencing, the cDNA sequence was also analyzed for fusions only. A pipeline following GATK Best Practices was used for bioinformatics analysis.

TMB was defined as the total number of mutations divided by the total coding region amplified and is reported as mutations/megabase (16). For SNVs and small indels, only the pathogenic/likely pathogenic variants were reported. Copy number alterations were identified based on read depths normalized to a pool of sex-matched normal samples (17).

### Statistical methods and software

The median overall survival (OS) was determined using Kaplan–Meier estimates with the survival and survutils packages in R, and integrated molecular information was visualized using the CoMut Python package (18). Mutational signatures were evaluated using the MutationalPatterns package in R (19). The contributions of known single-base substitution (SBS) COSMIC signatures (version 3.3) to the mutational profiles in each sample were determined using strict signature refitting. To reduce signature misattribution, bootstrapping was performed with 1,000 iterations to verify the stability of the refitting using the “fit_to_signatures_bootstrapped” function with “method” = “strict,” “n_boots” = 1000, and other options at default. Stable evidence of the presence of a signature in a sample was inferred from the percentage of iterations in which the signature was found (contribution >0), and a signature was considered stable if present in >50% of iterations. The relative contribution of a signature to a mutational profile was defined as the number of mutations due to the signature relative to the total number of assigned mutations in an iteration. Determination of the predominance of a stable signature was based on its mean relative contribution in the 1,000 iterations. Indel signatures were not further analyzed due to limited events.

## Results

### Patient and disease characteristics

We identified four cases with thoracic SMARCA4-dUT within the study period, including three men and one woman. A detailed description of the clinical characteristics is presented in **Table 1**. The median age was 61.5 years (range, 49–72 years). All cases had a smoking history, with a median pack per year (ppy) of 14 (range, 1–35 ppy). In one case, the tumor primarily involved the mediastinum, while the primary tumor in the three other cases was in a lung lobe(s). The median tumor size in the greatest dimension was 7.6 cm (range, 3.7–12 cm). Two cases (nos. 1 and 3) presented with early-stage disease (stages IIIa and IIb, respectively), while the disease was at the late stage in the remaining two cases (stages IIIb and IV). The sites of metastatic disease included the lymph nodes (mediastinal and cervical) and the thyroid. Case 3 presented with asymptomatic disease detected on routine surveillance for lung cancer, although the patient had a large pneumothorax and mediastinal shift on imaging. All others were symptomatic. The radiologic findings are described in **Table 1**.

**Table 1:** Clinical features of cases with SMARCA4 deficient undifferentiated thoracic tumor.

Case ID	Age at Dx	Sex	Presentation and ECOG PS	Medical Hx	Smoking Hx (PPY)	Radiology	Stage	Management	Status	Time from Dx (Days)	Relapse/Progression
1	61	F	Routine ILD screening ECOG:1	Autoimmune mediated ILD	8ppy	Right lung base mass (3.3cm) and subcarinal nodal mass (3.7 cm)	IIIa	Carboplatin and pemetrexed	Alive with PD	241	Progressed 6 mo after dx with metastasis to iliac bones. Radiotherapy to iliacs and started on tazemetostat
2	62	M	Dyspnea and neck swelling ECOG: 3	Schizophrenia	20ppy	Superior mediastinal mass (10cm) with compression of BCA and SVC, and conglomerate lymphadenopathy in mediastinum, right hilum, and upper abdomen	IV	Palliative local radiotherapy	Deceased	34	NA
3	72	M	Lung cancer screening ECOG:1	Bladder cancer	35ppy	Irregularly lobulated 5.2cm left lower lobe mass. Large left-sided pneumothorax and mild degree mediastinal shift	IIb	Lobectomy + Carboplatin/Cisplatin and pemetrexed	Alive	411	No
4	49	M	Dyspnea, chest pain and weight loss ECOG: 3	Emphysema	1ppy	Right upper lobe mass (12cm), broadly abutting the pleura and eroding the adjacent ribs. Severe bilateral emphysema of background lung. Hypermetabolic thoracic lymph nodes	IIIb	Comfort care	Deceased	125	NA

BCA, brachiocephalic artery; Dx, diagnosis; F, female; Hx, history; ILD, interstitial lung disease; M, male; mo, month; NA, not applicable; PD, progressive disease; PPY, packs per year; SVC, superior vena cava. ECOG PS, Eastern Cooperative Oncology Group performance status.

### Treatment and outcomes

**Figure 1:**
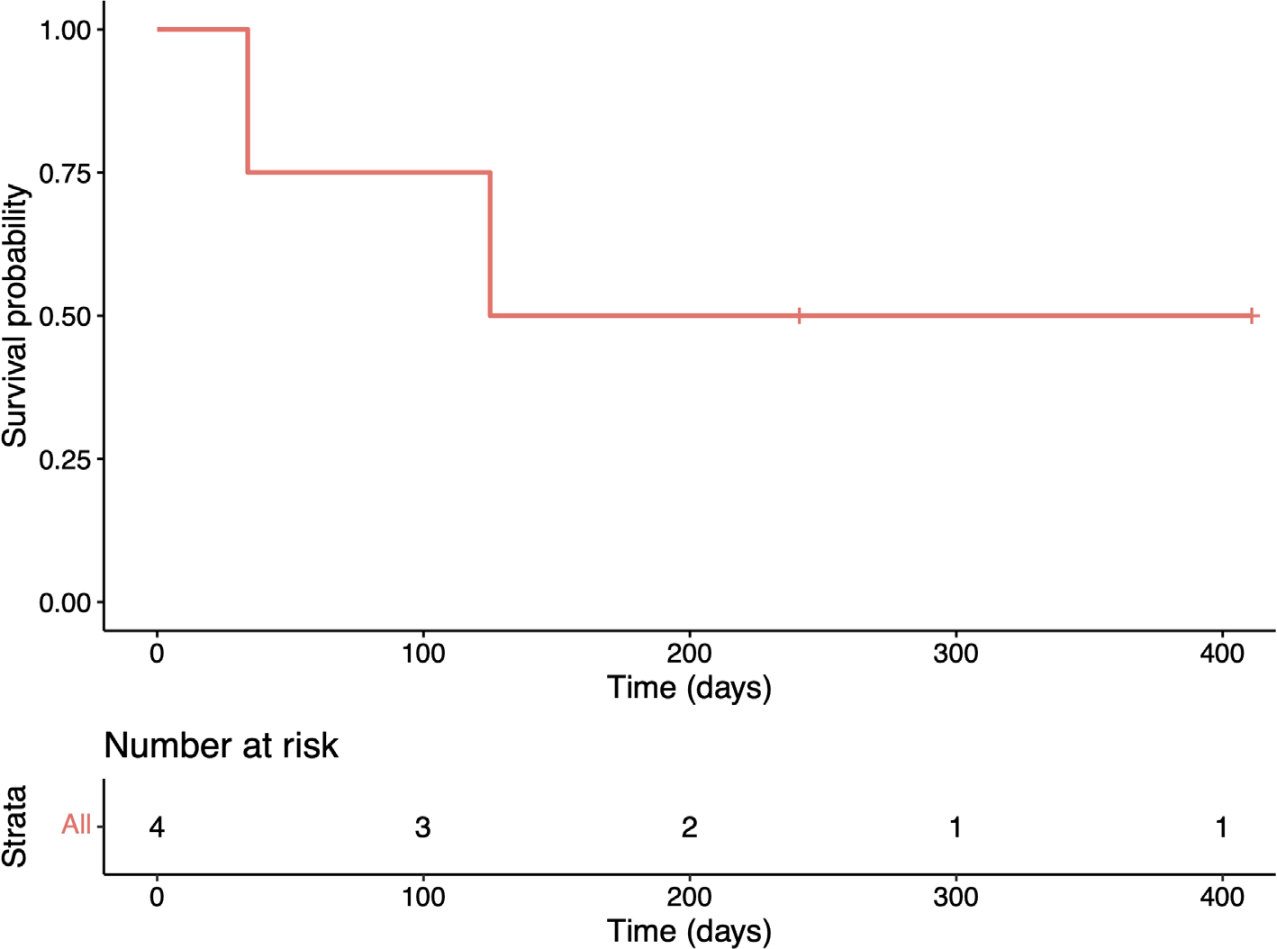
Kaplan–Meier plot showing the overall survival of the cohort.

Two cases (nos. 1 and 3) received therapy with curative intent, while the other two received palliative therapy, including local radiotherapy to the mediastinal mass in case 2, and comfort measures (**Table 1**). For the two cases treated with curative intent, both received standard chemotherapy with cisplatin/carboplatin and pemetrexed. Case 1 showed disease progression and metastasis to the iliac bones that required bilateral radiotherapy to the iliacs and tazemetostat treatment. Follow-up data on the response to tazemetostat were unavailable at the time of writing the manuscript. Case 3 had a lobectomy and adjuvant chemotherapy, with no evidence of relapse, 411 days after the initial diagnosis. Two patients (those managed palliatively) died of disease, with a median OS of 4.1 months (95%CI = 1.1–not estimable) after a median follow-up of 183 days (**Table 1**; **Figure 1**).

### Morphology and immunophenotype

**Figure 2:**
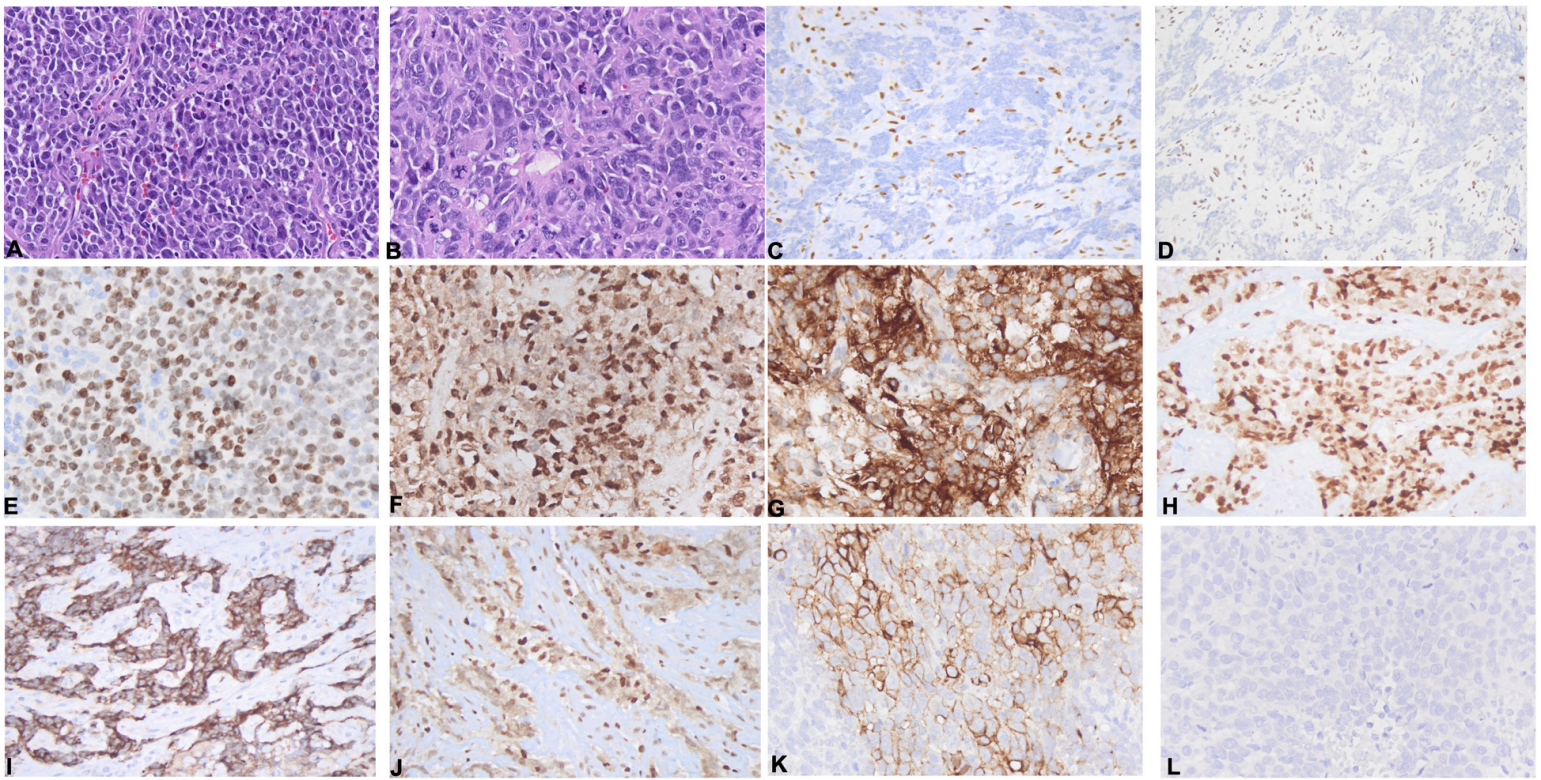
Morphologic and immunophenotypic features. **(A, B)** Sections of thoracic SMARCA4-deficient undifferentiated tumor (SMARCA4-dUT) showing dyscohesive cells **(A)**, a plasmacytoid morphology **(B)**, and frequent mitoses and occasional tumor giant cells **(B)**. **(C–K)** Immunohistochemical panel showing loss of BRG1 **(C)** and BRM **(D)** in neoplastic cells with preserved expression in lymphocytes and the expression of SALL4 **(E)**, FLI1 **(F)**, CD10 **(G)**, and WT1 **(H)** and variable neuroendocrine markers, including a case with synaptophysin expression **(I)** with retained RB expression **(J)** and lack of expression of INSM1 **(L)**, as well as a different case with CD56 expression (**K**).

The tumors demonstrated diffuse sheets of dyscohesive neoplastic cells on histologic sections and lacked morphologic evidence of squamous or glandular differentiation in all cases (**Figure 2A**). The neoplastic cells exhibited a largely monotonous epithelioid appearance with variable plasmacytoid (**Figure 2B**) and rhabdoid features. Interspersed multinucleated tumor giant cells were observed in patient 1 (**Figure 2B**). Moderate to extensive areas of necrosis were seen in cases 3 and 4. The immunophenotypic features are described in **Table 2**; **Figures 2C–L**. All examined cases demonstrated loss of nuclear BRG1 expression (**Figure 2C**) in the neoplastic cells, retained INI expression, and loss of BRM expression (in three out of three cases) (**Figure 2D**). NUT expression was uniformly absent. Pan-cytokeratin, claudin-4, and CAM5.2 were absent in all cases. The markers of lung carcinoma, such as TTF1, CK7, p40, and p63, showed absent or only focal weak expression (**Supplementary Figure S1**). Among the tested markers of stemness, SALL4 was positive in two of the four cases (**Figure 2E**), SOX2 in two of four cases, and CD34 was negative in all four cases. FLI1 was positive in three of three cases (**Figure 2F**), and CD10 was positive or patchy positive also in three of three cases (**Figure 2G**). WT-1 was expressed in a single case (out of three cases) (**Figure 2H**). Regarding the neuroendocrine-related markers, all cases expressed synaptophysin to varying degrees (**Figure 2I**). Chromogranin was absent in all cases, and nuclear RB was retained in three out of three cases (**Figure 2J**). CD56 (**Figure 2K**) was expressed in one of the four cases, and INSM1 expression was absent in three out of three cases (**Figure 2L**). Other lineage-specific markers were negative. The potential diagnostic pitfalls resulting from the patterns of expression of these antigens are discussed in **Supplementary Table S2**.

**Table 2:** Immunohistochemical features of patients with SMARCA4 deficient undifferentiated thoracic tumor.

Patient ID	BRG1	BRM	INI	CD34	SOX2	SALL4	CD10	FLI1	OCT4	Synapto	Chromo	CD56	INSM1	RB	PanCK	Claudin	CAM5.2	CK7	TTF-1	p63	p40	CK5	CK20	NUT1	Calretinin	WT1
1	lost	lost	retained	–	–	+	+	+	–	+ (patchy)	–	–	–	retained	–	ND	–	–	+ (patchy)	ND	–	–	–	–	–	–
2	lost	ND	retained	–	–	–	+	+	–	+ (weak)	–	–	–	retained	–	–	–	–	–	+ (focal and very weak)	ND	–		–	–	–
3	lost	lost	retained	–	+ (focal)	+	ND	ND	ND	+	–	+	ND	ND	–	ND	–	–	–	–	–	–	–	–	ND	ND
4	lost	lost	retained	–	+ (weak, patchy)	–	+	+	–	+	–	–	–	retained	–	–	–	–	–	ND	–	ND	–	–	–	+

Chromo, chromogranin; synapto: synaptophysin; PanCK, pancytokeratin.

### Genomics

**Figure 3:**
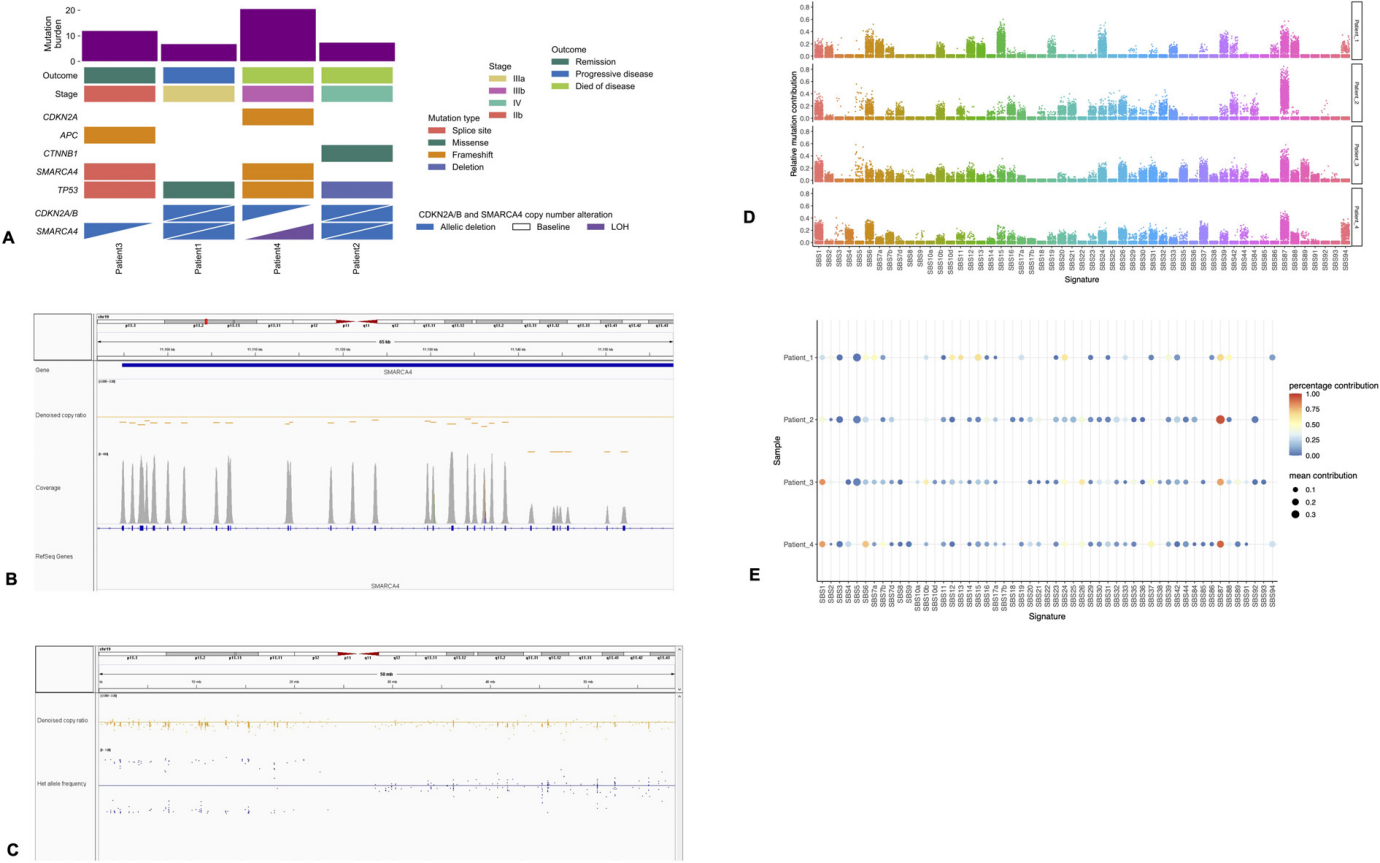
Genomic features. **(A)** Integrated molecular information plot showing the mutations, tumor mutation burden, disease stage, and outcomes. **(B)** Snapshot of Integrative Genomics Viewer (IGV) illustrating homozygous (deep) deletion involving SMARCA4, demonstrated by a significant diminution of the read coverage **(C)** copy neutral loss of heterozygosity involving the short arm of chromosome 19. The copy ratio is normal along the entire chromosome (*orange dots*). However, the short arm of the chromosome shows either A or B alleles, but not both (*blue dots on either side of the center*, but not on the *center*). **(D)** Relative contributions of the single-base substitution (SBS) signatures to the mutational profiles using strict COSMIC signature refitting with 1,000 iterations. **(E)** Balloon plot highlighting the predominance of the COSMIC SBS signature SBS87. Percentage contribution refers to the proportion of 1,000 iterations where a signature of interest was detected (contribution >0). Higher percentages indicate stable or significant evidence of the signature in a sample. There is weak to no evidence of contribution by the smoking-related signatures SBS4 and SBS92 to the mutational profiles. Mean contribution is the mean of the percentage of mutations within a sample attributed to the signature across the 1,000 iterations.

The median TMB was 10 mutations/MB. All of the tumors were microsatellite-stable. Each case had a pathogenic *TP53* variant (**Figure 3A**). Other pathogenic variants were present only in single patients, including pathogenic *APC*, *CDKN2A*, and *CTNNB1* variants (**Figure 3A**). The patient with limited disease and durable response had a TMB of 12 mutations/MB and pathogenic *APC* variant (**Figure 3A**). There were no pathogenic *EGFR*, *MET*, and *KRAS* variants identified, and *ALK*, *ROS1*, *MET*, or *BRAF* fusions were not observed in any of the patients. Apart from case 3, all other cases showed loss of *CDKN2A/B*, with two cases displaying deep deletions and the other displaying a frameshift mutation of *CDKN2A* accompanied by monosomy 9.

#### Genomic basis of loss of *SMARCA4*

In two cases, the *SMARCA4* deficiency was found to be due to homozygous (deep) deletions involving the *SMARCA4* gene (**Figures 3A, B**). For case 3, there was monosomy 19, resulting in loss of one copy of the *SMARCA4* gene, and a splice site mutation involving the other allele, together leading to bi-allelic loss of the gene product. In case 4, a pathogenic frameshift variant of *SMARCA4* was accompanied by copy neutral loss of heterozygosity involving the short arm of chromosome 19 (**Figure 3C**). No molecular alterations of SMARCA2 were identified in any of the cases. The complete list of genomic findings, including SNVs, indels, and copy number changes, is illustrated in **Supplementary Table S3**.

#### Mutational signatures

Mutational signature analysis revealed SBS87 to be stably present in all patients (**Figures 3D, E**). The cosine similarity between this COSMIC signature and the observed mutational profiles in our samples ranged from 0.5 to 0.8, consistent with moderate to high similarity. It was the most dominant signature contributing to SBS in three of four patients and the second most dominant in the remaining patient, with an overall range of mean relative contributions of 13.4%–36.5% of SBS (**Figures 3D, E**). The tobacco-related SBS signatures, SSB4 and SSB92, were not stably present in any of the patients and were not predominant contributors of the observed SBS mutational profiles (mean relative contribution, 0%–3.3%) (**Figures 3D, E**).

## Discussion

SMARCA4-dUT is a recently defined entity with aggressive behavior, typically presenting with extensive disease and unfavorable prognosis. Description of the landscape of the clinicopathologic and genomic features of this relatively new entity is evolving. In this mini-series, we described four cases seen in our institution, highlighting novel phenotypic findings and potential variability in the genomic and clinical features.

The patient with typical thoracic SMARCA4-dUT is middle-aged with a significant history of smoking, presenting with advanced disease. All of our cases had significant smoking history, but our series showed a later age at presentation (61.5 years) compared with the median aggregated by Perret et al. from three previous series (48 years, range = 27–90 years) (6). We observed bulky mediastinal disease in one case and lung-based disease in three others, with variable local spread. Among the latter was a case (no. 3) with asymptomatic disease detected on lung cancer surveillance. Case 3 is an example of the emerging reports of patients presenting with limited disease including those amenable to resection (20, 21).

The prognosis of thoracic SMARCA4-dUT is poor, with a median OS of 4–7 months (2, 6–8). The median OS in our cohort was 4.1 months, which is comparable to the survival estimates from larger studies. Luo et al., however, have described a small series of patients with early-stage thoracic SMARCA4-dUT with a median OS of 15.6 months (21). There are reports of patients with operable early-stage disease or disease rendered resectable after neoadjuvant immune checkpoint blockage (11, 20). Our cohort included a case with asymptomatic early-stage disease and no evidence of disease 13 months after treatment. Variable responses to checkpoint inhibition have also been documented, including in patients with durable response (10–15). Our findings and those of others altogether depict the possibility of variability in the prognosis and that early-stage disease may not be as uncommon and may have better prognosis.

The hallmark diagnostic features of thoracic SMARCA4-dUT are the lack of differentiation by morphological and immunohistochemical evaluation, loss or significantly decreased BRG1 and BRM (SMARCA2) expression in neoplastic cells, and expression of stemness markers. BRG1 expression was absent in all our cases. BRM was absent in all tested cases. Epithelial markers and the markers of non-small cell lung cancer (NSCLC), such as pan-cytokeratin, CAM5.2, claudin-4, CK5, p40, and CK7, in all tested cases were not expressed, and single cases showed focal weak TTF-1 and p63 expression. There was variable stemness marker expression including SALL4 and SOX2. FLI1 expression was observed in three out of three cases, a finding of crucial diagnostic significance. FLI1 is a transcription factor in the ETS family expressed in Ewing sarcoma and angiosarcoma, as well as in an increasing list of neoplasms. In NSCLC, FLI1 was found to be a marker of worse prognosis (22). To the best of our knowledge, the expression of FLI1 in thoracic SMARCA4-dUT has not been reported, and awareness of the possibility of FL1I expression is essential in the differential diagnosis of high-grade or undifferentiated malignant neoplasms, particularly in the presence of CD34, one of the stemness markers expressed in SMARCA4-dUT, as these markers are also co-expressed in angiosarcoma. The frequency and the prognostic impact of FLI1 expression in thoracic SMARCA4-dUT need to be explored in large studies. Evaluation of the neuroendocrine markers in thoracic SMARCA4-dUT is relevant considering the differential diagnosis including neuroendocrine carcinoma, which has high mitotic activity and necrosis, similar to thoracic SMARCA4-dUT. Synaptophysin, a less specific neuroendocrine marker, is not infrequently expressed in thoracic SMARCA4-dUT, further confounding diagnostic evaluation (4, 6, 7). However, chromogranin and CD56, other neuroendocrine markers, are generally negative in thoracic SMARCA4-dUT (6, 7). Synaptophysin expression was present in all our cases, while chromogranin was absent. Of note is that a single case co-expressed CD56, presenting another potential pitfall in limited immunohistochemical marker panels. All cases showed a lack of expression of INSM1, a more recent marker of neuroendocrine differentiation with reportedly superior performance in the diagnosis of thoracic neuroendocrine tumors (23). When dealing with limited diagnostic tissue, in the appropriate clinicopathologic context, INSM1 could be essential as a stand-alone marker for excluding a neuroendocrine neoplasm. The expression of WT-1, observed in a single tested case, also presents a potential diagnostic challenge in differentiation from a malignant mesothelioma, in particular in a pleural-based disease. However, CK5 and calretinin, other markers positive in malignant mesothelioma, were not expressed, highlighting the essence of selective extended panels to ensure that the correct diagnosis is made (24). A summary of pertinent diagnostic pitfalls is highlighted in **Supplementary Table S2**.

SMARCA4 deficiency predominantly results from bi-allelic loss of *SMARCA4* through various genetic mechanisms such as frameshift, nonsense, and splice site mutations, deletions, and copy neutral loss of heterozygosity. We observed biallelic loss of *SMARCA4* through some combinations of these mechanisms. In addition to these genetic lesions, the underlying loss of one SMARCA4 allele in a single case was monosomy 19. Chromosome-level abnormalities leading to SMARCA4 loss are, however, thought to be rare. Molecular profiling of thoracic SMARCA4-dUT has been reported to show the presence of tobacco/smoking-related mutational signature, pathogenic variants in *TP53, STK11, KEAP1, and KRAS*, and a high TMB (4, 6, 7). The high median TMB of 10 mutations/MB and the presence of *TP53* variants in all of our patients mirror the findings from prior studies. However, evidence of the stable presence and dominant contributions of tobacco-related COSMIC SBS signatures was not observed in our series. Instead, the predominant SBS signature was the SBS87 signature that is similar to the mutational profile induced by exposure to thiopurine chemotherapy treatment (25). The SBS87 signature has been shown to be associated with durable response to immunotherapy in advanced NSCLC and presents a candidate biomarker for the prediction of response to immune checkpoint inhibition (26). The lack of evidence of the dominant contributions of smoking-related SBS mutational signatures, in spite of the significant smoking history, high TMB, and pathogenic *TP53* variants, could be attributed to our use of a targeted next-generation sequencing (NGS) panel with limited representativeness of the detected mutational processes or may have resulted from a genuine underlying biologically inspired process. Of note is that, using a targeted NGS panel of a similar size to ours, Rekhtman et al. found the smoking signature in most but not all of their cohort of SMARCA4-dUT cases, where genomic testing was performed (4). In addition, in other tobacco-related lung cancers with comparable TMB to ours, targeted NGS panels have detected these signatures (27). However, a proportion of lung cancer patients with substantial smoking history lack evidence of smoking-related mutagenesis signatures on whole-exome sequencing, suggesting a smoking-independent initiation of carcinogenesis (28). The significance of our findings is thus unclear and underscores the need for larger studies, preferably with exome- or genome-level analysis to verify these findings. Pathogenic/likely pathogenic variants in *CTNNB1, APC*, and *CDKN2A* were also detected. The prognostic role of these variants in thoracic SMARCA4-dUT is unknown. *CDKN2A* is one of the frequently mutated genes in this entity (9). Loss of *CDKN2A* was observed in all our cases, except in the long-term disease-free survivor. Lung cancer patients with wild-type *CDKN2A* are less likely to experience disease progression following therapy compared with those with *CDKN2A* loss (29). Exploration of any association of lack of altered *CDKN2A* with the extent of disease and response to therapy in thoracic SMARCA4-dUT would require large studies.

The main limitation of this study is the small number of patients included, precluding definite identification of subgroups of patients with statistically significant differences in clinicopathologic and genomic features. The single-institutional nature of the study, in addition to contributing to the small sample size, also adds to the inherent limitations from experiences from a single institution. Larger, preferably multi-institutional studies will have the statistical power to determine whether any such subgroups exist and also determine the degree of generalizability of our findings.

In summary, we have comprehensively described a series of thoracic SMARCA4-dUT cases including an atypical case with limited disease and with durable response and that lacked *CDNK2A* alteration. We highlighted novel phenotypic findings such as the expression of FLI1, WT-1, and CD56 and their potential for creating diagnostic pitfalls. In addition, we identified genomic signatures suggesting the possible contributions of non-smoking-related processes in carcinogenesis, which require confirmation in larger studies with genome/exome-level interrogation.

## Data Availability

The original contributions presented in the study are included in the article/**Supplementary Material**. Further inquiries can be directed to the corresponding authors.

## Supplementary material

Supplementary Figure 1 Extended panel of immunostains with patient ID as rows and tested antibodies as columns. Empty spaces indicate antibodies that could not be tested for in corresponding patients. Image on row 1 column 3 is TTF and row 2 column 3 is p63.
