# Supplementary material for: Updating the genomic and clinicopathologic features of thoracic SMARCA4-deficient undifferentiated tumor: a mini-series including a long-term survivor
Source: Front Oncol. 2025 Aug 20;15:1601443. doi: 10.3389/fonc.2025.1601443 (PMC12405327; doi:10.3389/fonc.2025.1601443)
Supplement: Supplementary file 1 [file DataSheet1.zip › Table 1.docx]

Supplemental Table 1: Detailed description of antibodies used for immunohistochemical staining

| **Antibody** | **Clone** | **Manufacturer** | **Dilution** |
| --- | --- | --- | --- |
| BRG-1 | Polyclonal | Abcam | 1:500 |
| INI-1 | 25/BAF47 | BD Bioscience | 1:400 |
| CD34 | QBEnd/10 | Leica Biosystems | RTU |
| SOX-2 | SP76 | Ventana | RTU |
| SALL4 | 6E3 | Ventana | RTU |
| CD10 | 56C6 | Leica | RTU |
| FLI-1 | MRQ-1 | Cell Marque | RTU |
| Oct-4 | MRQ-10 | Ventana | RTU |
| Synaptophysin | SP11 | Ventana | RTU |
| Chromogranin | LK2H10 | Ventana | RTU |
| CD56 | CD564 | Leica | RTU |
| INSM1 | MRQ-70 | Cell Marque | RTU |
| RB | 13A10 | Leica | 1:100 |
| Pan CK | AE1/AE3 | Leica | RTU |
| Claudin-4 | 3E2C1 | Biocare | RTU |
| CK Cam 5.2 | Cam5.2 | Ventana | RTU |
| CK7 | RN7 | Leica | RTU |
| TTF-1 | 8G7G3/1 | Ventana | RTU |
| P63 | 4A4 | Ventana | RTU |
| P40 | BC28 | Ventana | RTU |
| CK5 | XM26 | Leica | RTU |
| NUT-1 | C52B1 | Cell Signaling | 1:100 |
| Calretinin | SP65 | Ventana | RTU |
| WT-1 | WT49 | Leica | RTU |

Abbreviations

RTU: Ready to use
