# Supplementary material for: Updating the genomic and clinicopathologic features of thoracic SMARCA4-deficient undifferentiated tumor: a mini-series including a long-term survivor
Source: Front Oncol. 2025 Aug 20;15:1601443. doi: 10.3389/fonc.2025.1601443 (PMC12405327; doi:10.3389/fonc.2025.1601443)
Supplement: Supplementary file 1 [file DataSheet1.zip › Table 2.docx]

Supplemental table 2: List of Entities Posing Diagnostic Pitfalls

| Diagnostic Entity | Clinico-radiologic Features | Morphologic Features | Typical Immunohistochemical | Potential pitfalls/New findings identified in this study |
| --- | --- | --- | --- | --- |
| SMARCA4-dUT | Usually presents with bulky disease of the thorax  Majority of patients are heavy smokers with genomic smoking signature  Uniformly aggressive behavior with poor prognosis | Diffuse sheets of discohesive epithelioid cells, vesicular chromatin, and prominent nucleoli  Lack of definite evidence of epithelial differentiation | BRG1 (SMARCA4) loss  BRM (SMARCA2) loss  Positive for markers of stemness (CD34, SALL4, SOX2)  Negative or only focal/weak positive for markers of epithelial differentiation | May present with limited disease.  The disease may have less aggressive course in some patients  Smoking signature is not uniformly seen in all patients in spite of history of smoking. |
| SMARCA4-deficient non-small cell lung carcinoma | Variable | Glandular or squamous differentiation | Loss of BRG1 and BRM, with expression of markers of epithelial differentiation (claudin-4, BerEP4 or MOC31, B72.3, CEA, CD15 (LeuM1), and BG8)  Negative for markers of stemness | Focal/patchy expression of markers of epithelial origin can be seen in SMARCA4 dUT |
| Malignant Mesothelioma | Diffuse pleural thickening, pleural effusion, associated with asbestos exposure | Epithelioid, sarcomatoid, or biphasic growth patterns | Immunohistochemical evidence of mesothelial origin - expression of a minimum of two mesothelial markers (WT1, D2-40, and CK5/6) and lack expression of epithelial markers (claudin-4, BerEP4 or MOC31, B72.3, CEA, CD15 (LeuM1), and BG8) | SMARCA4-duT may also be pleural based.  WT-1, a mesothelial marker was detected in 1 of our cases. Expanded cocktail including additional mesothelial markers and BRG1 required to avoid pitfall |
| Neuroendocrine Carcinoma | Usually peripherally located, and presents with rapid growth | Variable growth patterns including sheets, organoid nesting, trabeculae, resetting and palisades. Associated with crush artifacts, and necrosis | Expression of neuroendocrine markers (synaptophysin, chromogranin, CD56, INSM1); BRG1 retained | Patchy synaptophysin expression as observed in our series, is known in SMARCA4 duT. Co-expression of synaptophysin and CD56 seen in one of our cases. INSM1 considered a more reliable marker of neuroendocrine differentiation was absent in all cases. |
| Angiosarcoma of thorax | Infiltrative proliferation, presenting with pleural thickening, effusion, hemothorax, and compressive symptoms. May be associated with radiation exposure | Variable degree of vasoformation ranging from anastomosing vessels to solid sheets of spindle or epithelioid tumor cells | Expression of endothelium associated markers (CD31, CD34, ERG, and FLI-1) | FLI-1 expression was present in # our cases, presenting a pitfall when interpreted in isolation. CD34 in addition to being endothelium associated, is a marker of stemness, and expressed in SMARCA4-duT. Co-expression of CD34 and FL1-1 suggestive of angiomatoid histogenesis, can be seen in SMARCA4-duT |
| SMARCA4-dUT | Usually presents with bulky disease of the thorax  Majority of patients are heavy smokers with genomic smoking signature  Uniformly aggressive behavior with poor prognosis | Diffuse sheets of discohesive epithelioid cells, vesicular chromatin, and prominent nucleoli  Lack of definite evidence of epithelial differentiation | BRG1 (SMARCA4) loss  BRM (SMARCA2) loss  Positive for markers of stemness (CD34, SALL4, SOX2) | May present with limited disease.  The disease may have less aggressive course in some patients  Smoking signature is not uniformly seen in all patients in spite of history of smoking. |

Legend: BRG: Brahma-related gene 1; INSM1: Insulinoma-associated protein 1; FLI1: Friend leukemia integration 1;
