# Supplementary material for: Updating the genomic and clinicopathologic features of thoracic SMARCA4-deficient undifferentiated tumor: a mini-series including a long-term survivor
Source: Front Oncol. 2025 Aug 20;15:1601443. doi: 10.3389/fonc.2025.1601443 (PMC12405327; doi:10.3389/fonc.2025.1601443)
Supplement: Supplementary file 1 [file DataSheet1.zip › Table 3.docx]

Supplementary Table 3: Complete genomic features of cases with SMARCA4 deficient undifferentiated thoracic tumor.

1. Single nucleotide variants and indels

| Patient ID | Gene | Nucleotide Change | Amino Acid Change | Variant Allele Fraction (%) |
| --- | --- | --- | --- | --- |
| 1 | TP53 | NM_000546.5:c.747G>T | p.Arg249Ser | 55.6 |
| 2 | TP53 | NM_000546.5:c.764_766del | p.Ile255del | 77.1 |
| 2 | CTNNB1 | NM_001904.4: c.121A>G | p.Thr41Ala | 41.8 |
| 3 | TP53 | NM_000546.5:c.672+1G>T | p.? (splice site variant) | 76.8 |
| 3 | SMARCA4 | NM_003072.4:c.3081+1G>T | p.? (splice site variant) | 53.1 |
| 3 | APC | NM_000038.6:c.7932_7935del | p.Tyr2645fs | 66.1 |
| 4 | TP53 | NM_000546.5:c.794dupT | p.Arg267fs | 75.1 |
| 4 | SMARCA4 | NM_003072.4:c.2582_2597del | p.Glu861fs | 72.0 |
| 4 | CDKN2A | NM_000077.4:c.134del | p.Gly45fs | 58.3 |

1. Copy Number Changes

| Patient ID | Copy Number Loss | Copy Number Gains |
| --- | --- | --- |
| 1 | Deep (Homozygous) deletion  MTAP  CDKN2A/B  SMARCA4 |  |
| 2 | Deep (Homozygous) deletion  MTAP  CDKN2A/B  SMARCA4 | FGFR1 |
| 3 | Deletion 5q  Deletion 6p21.33 involving exon 5 of HLA-DRB1  Deletion 8p23.2-p12  Deletion of 9q  Deletion 13q  Deletion 14q  Deletion 18q21.1-q23  Monosomy 19, notably leading to loss of SMARCA4  Deletion 20p  Monosomy 22 | Gain of 8p11.23-8q  Gain of 10p  Gain of 11q |
| 4 | Deletion 9p24.2-21.1 | Gain of 1p36.31-34.3  Gain of 1q21.1-44  Gain of 3q12.1-29  Gain of 5p15.32-12  Gain of 7p22.2-11.2  Gain of 8p11.23-p1.21  Gain of 10p15.2-11.21  Trisomy 20 |
