# Supplementary figures and images for: Updating the genomic and clinicopathologic features of thoracic SMARCA4-deficient undifferentiated tumor: a mini-series including a long-term survivor
Source: Front Oncol. 2025 Aug 20;15:1601443. doi: 10.3389/fonc.2025.1601443 (PMC12405327; doi:10.3389/fonc.2025.1601443)

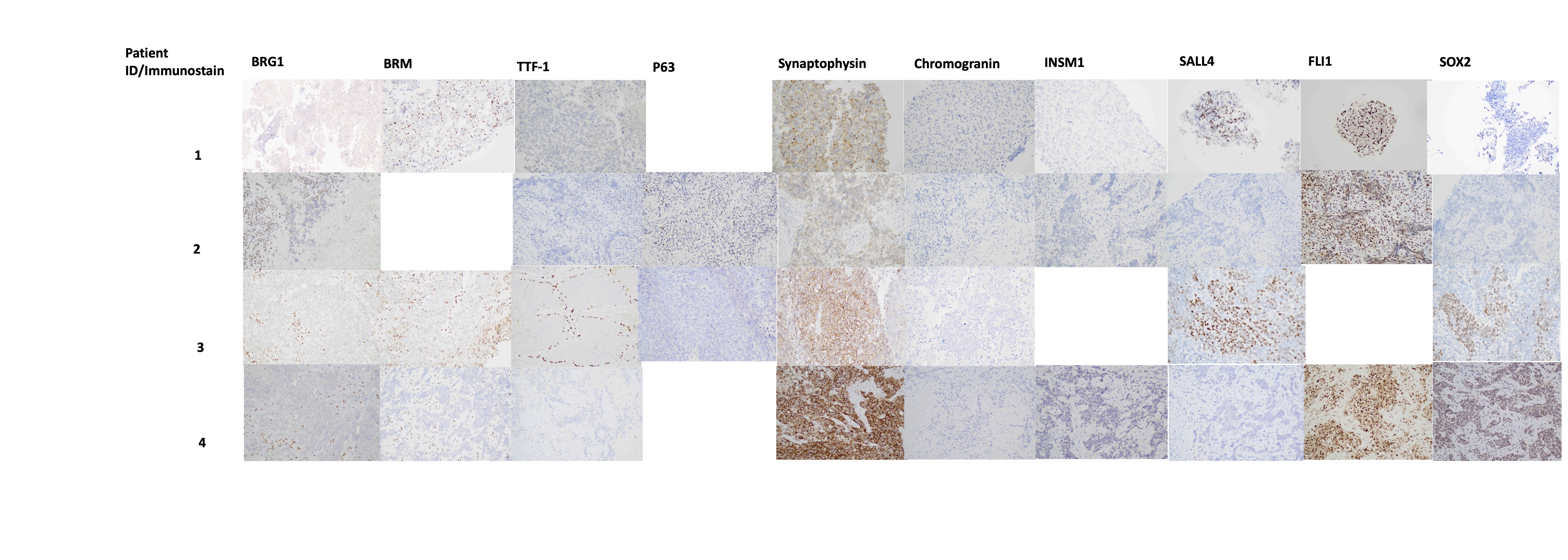

Supplement: Supplementary Figure 1 — Extended panel of immunostains with patient ID as rows and tested antibodies as columns. Empty spaces indicate antibodies that could not be tested for in corresponding patients. Image on row 1 column 3 is TTF and row 2 column 3 is p63. [file Image1.jpg]
